# Visual versus automated CT perfusion for outcome prediction in anterior circulation stroke treated with mechanical thrombectomy

**DOI:** 10.2478/raon-2026-0039

**Published:** 2026-07-29

**Authors:** Igor Rigler, Tina Gspan, Alja Longo, Janja Pretnar Oblak

**Affiliations:** 1 Clinical Department of Vascular and Intensive Neurology Therapy, Neurology Clinic, University Medical Centre Ljubljana, Ljubljana, Slovenia; 2 Clinical Institute of Radiology, Institute of Neuroradiology, University Medical Centre Ljubljana, Ljubljana, Slovenia; 3 Department of Neurology, Faculty of Medicine, University of Ljubljana, Ljubljana, Slovenia

**Keywords:** CT perfusion, acute stroke, mechanical thrombectomy, prognosis

## Abstract

**Background:**

The prognostic value of simple visual assessment of CT perfusion (CTP) for predicting clinical outcomes has already been demonstrated in stroke patients undergoing mechanical thrombectomy (MT). Automated software, however, has become the dominant method of CTP evaluation. The aim of the study was to compare the prognostic accuracy of visually graded CTP maps with automated CTP analysis on clinical outcomes in anterior circulation stroke patients treated with MT.

**Patients and methods:**

In the single centre we retrospectively analysed consecutive patients with anterior circulation large-vessel occlusion treated with MT. Baseline imaging included non-contrast brain CT, CT angiography (CTA) with collateral status (CS) and CTP. Time to peak (TTP) maps were visually graded on a four-level ordinal scale based on estimated penumbra. Automated CTP was processed with syngo.via (SYV) using default thresholds and classified on a comparable ordinal scale. The primary endpoint was favourable 90-day functional outcome (Rankin Scale (mRS) ≤ 3).

**Results:**

We included 579 patients. Agreement between visual and automated CTP was fair (κ = 0.36). Both methods were significantly associated with favourable outcome (p < 0.01), with visual grading showing a slightly stronger association (Pearson χ^2^ = 30 vs. 22). In multivariable models, visual CTP remained an independent predictor, whereas automated CTP lost significance when CS was included.

**Conclusions:**

Visual CTP grading provided a slightly better prognostic performance than automated CTP. Visual CTP assessment is a simple, accessible and clinically meaningful tool for both treatment decision making and outcome prediction in anterior circulation stroke treated with MT.

## Introduction

CT perfusion (CTP) is increasingly used in acute ischemic stroke workflow to estimate infarct core and salvageable penumbra. Automated CTP gained importance after DAWN and DEFUSE-3 trials established its role in selecting patients for mechanical thrombectomy (MT) beyond 6 hours and EXTEND trial demonstrated its utility for extending intravenous thrombolysis up to 9 hours or after wake-up stroke.^1-3^ More recently, RESCUEJapan LIMIT, SELECT2 and ANGEL-Alberta Stroke Program Early CT Score (ASPECTS) trials broadened MT eligibility to patients with large estimated ischemic cores using either automated CTP-derived core volumes or markedly reduced ASPECTS, though outcomes remained considerably poorer than in the small core patients.^4-6^

CTP therefore contributes not only to treatment selection but also enables prognostic assessment, particularly in patients with advanced age, frailty or significant comorbidities. However, automated CTP maps vary substantially between software platforms, reflecting differences in acquisition assumptions, deconvolution models and perfusion thresholds.^7^ Comparative studies show only moderate agreement in core and penumbra estimation and limited spatial concordance with final infarction^8-10^, raising concerns about the consistency of automated CTP for prognostication.

Visual CTP assessment remains widely used because it is rapid, easily interpretable and independent of software-specific thresholds. Time-to-peak (TTP) maps, in particular, provide a clear contrast between normal and critically delayed perfusion, while TTP »black-hole« regions have been linked with severely impaired hemodynamics.^11^ We previously demonstrated that a semi-quantitative TTP grading scale independently predicted 90-day outcome in MT treated anterior circulation stroke.^12^

Nevertheless, direct comparisons between visual and automated CTP remain limited. Given the dominance of automated analysis in clinical trials, evaluation of whether visual assessment retains prognostic impact compared to automated analysis is clinically relevant. This study compares visual TTP grading with automated CTP analysis using syngo.via (SYV) in a large, real-world cohort of anterior-circulation stroke patients treated with MT.

## Patients and methods

### Study design and patient selection

We performed a retrospective, single-centre observational study of consecutive patients with anterior circulation large-vessel occlusion (LVO) stroke treated with MT between January 2013 and December 2020. All patients were managed according to contemporary international stroke guidelines and were discussed within the hospital multidisciplinary stroke team.

Patients were included if they had an anterior circulation LVO (intracranial internal carotid artery, M1 or proximal M2 segment of the middle cerebral artery) on baseline CT angiography (CTA), underwent MT and had a documented 90-day modified Rankin Scale (mRS). We excluded patients with posterior circulation stroke and isolated distal medium-vessel occlusions. Separate models were constructed for visual and automated CTP, each including only patients with available respective perfusion data.

The National Medical Ethics Committee of Slovenia approved the study (ethical approval number: 0120-354/2019/4). The study adhered to the Declaration of Helsinki.

### Imaging protocol

Baseline imaging included non-contrast CT (CT), CTA, and CTP as part of the standard acute ischemic stroke imaging protocol. Imaging parameters and scanner hardware remained stable over the study period. Alberta Stroke Program Early CT Score (ASPECTS) was scored on CT. CTA was used to determine occlusion site and collateral status. CTP covered the supratentorial brain and was performed immediately after CTA.

Radiological studies were performed using a 40-row spiral CT unit (Somatom Sensation Open 40, Siemens Healthineers, Erlangen, Germany). Perfusion CT (80 kVp, 209 mAs) consisted of a 40-second series with 1 rotation/s during intravenous administration of iodinated contrast media. We injected 40 ml of nonionic low-osmolar iodinated contrast iopromide (370 mg/l, Ultravist; Bayer Healthcare, Leverkusen, Germany) antecubitally at a flow rate of 6 ml/s, followed by a 40-ml saline flush. Four 5-mm-thick slices were selected above the orbits at the level of the basal ganglia and the third ventricle. CTA was performed by injecting 80 ml of contrast media at a rate of 5 ml/s, followed by a craniocaudal scan from the vertex to the aortic arch with scanning parameters 120 kV, 150 mAs, gantry rotation time 0.5 s, collimation 40 × 0.6 mm, pitch 1.2 and field of view (FOV) 200 mm. Sections were reconstructed at 0.75 mm with a reconstruction increment of 0.4 mm. Additionally, maximum intensity projection (MIP) images in the coronal and axial planes were produced.

### Visual CT perfusion (CTP) assessment

**FIGURE 1.**
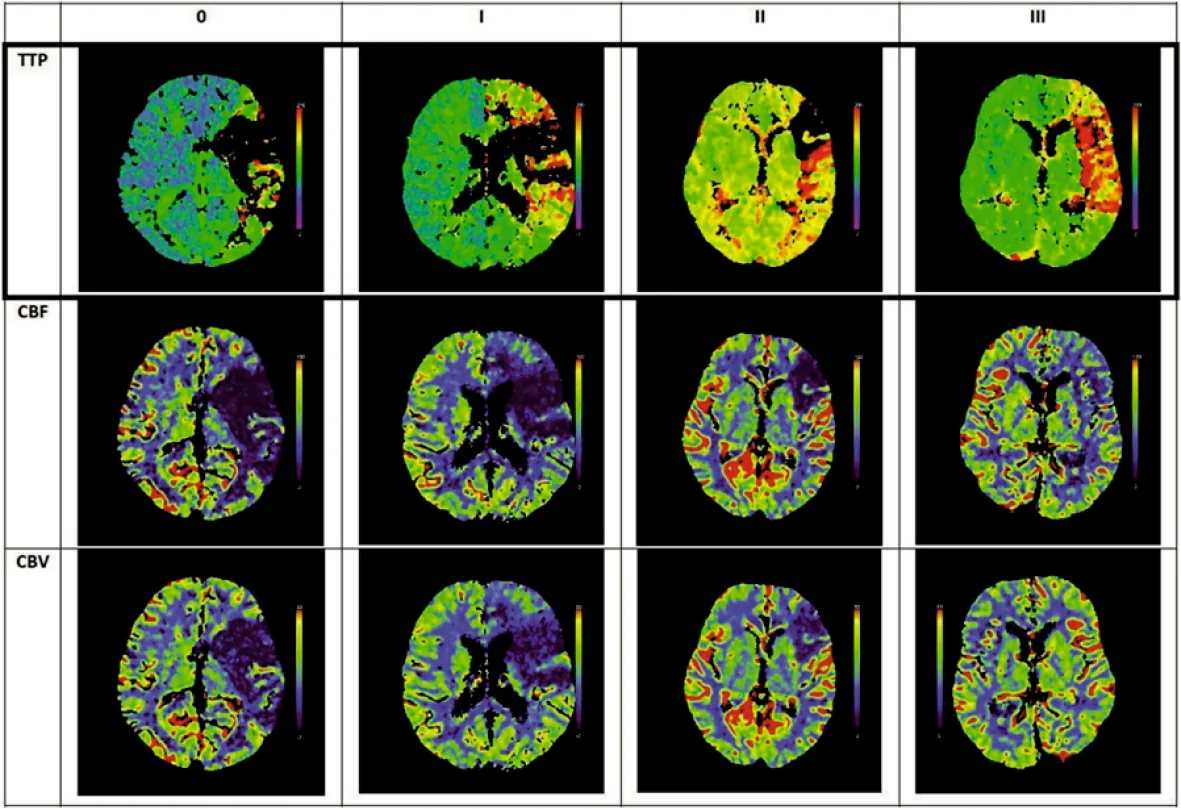
Examples of time to peak (TTP) based CT perfusion (CTP) maps divided into four categories, according to the percentage of »black hole« estimation in the hypoperfused region, with their corresponding cerebral blood flow (CBF) and cerebral blood volume (CBV) perfusion images.

Visual evaluation focused on time-to-peak (TTP) maps reviewed independently by a single reader with specialization in neuroradiology, with at least 5 years of clinical experience, who was blinded to clinical outcomes. Hypoperfusion was graded on a four-level ordinal scale reflecting the estimated proportion of penumbra within the affected vascular territory (0–25%, 25–50%, 50–75%, 75–100%) (Figure 1). Grading was based on the extent of markedly delayed - »black-hole« - TTP signal. This variable is referred to as *visual CTP*.

### Automated CT perfusion (CTP) analysis

Automated perfusion analysis was performed using SYV CT Neuro Perfusion (Siemens Healthineers, version VB40) with unchanged default settings throughout the study. To align with visual assessment, we derived a four-level ordinal measure representing the perfusion abnormality within the affected territory (0–25%, 25–50%, 50–75%, 75–100%). This variable is referred as *automated CTP*.

### Collateral assessment

Collateral status (CS) was evaluated on baseline CTA using a three-category visual scale adapted from established collateral grading systems.^13^ Collaterals within the affected vascular territory were classified as absent or minimal, reduced or normal/good compared with the contralateral hemisphere.

### Clinical data and outcome measures

Baseline variables included age, sex and prestroke functional status. Treatment-related variables included the use of IVT prior to MT and time from symptom onset (or last known well) to groin puncture. Recanalization was assessed using the Thrombolysis in Cerebral Infarction (TICI) scale; successful recanalization was defined as TICI ≥ 2b. Routine follow up CT was performed 24 hours after MT.

Functional outcome at 90 days was assessed using the mRS, obtained from outpatient visits, telephone interviews or medical records. Although 47% of patients achieved mRS ≤ 2 at 90 days, which is regarded as excellent outcome, the number of patients with mRS ≤ 2 within individual CTP subgroups was insufficient to allow stable modelling. Therefore, mRS ≤ 3 at 90 days was predefined as primary endpoint for favourable functional outcome.

### Statistical analysis

Continuous variables are reported as mean ± SD, categorical variables as counts and percentages.Group comparisons between patients with favourable (mRS ≤ 3) and unfavourable (mRS > 3) outcomes were performed using χ^2^ or Fisher’s exact test for categorical variables. Agreement between visual CTP and automated CTP ordinal classifications was assessed using Cohen’s κ coefficient.

To evaluate the independent prognostic value of visual and automated CTP, we constructed separate multivariable logistic regression models with favourable 90-day outcome (mRS ≤ 3) as the dependent variable. Two sets of models were used for each CTP technique:

*Baseline model (without CS):* age, baseline ASPECTS, IVT, time from symptom onset to *groin puncture, recanalization success (TICI ≥ 2b vs < 2b), and the respective CTP grade*.

*Extended model (with CS):* all base variables plus CS.

Odds ratios (ORs) with 95 % confidence intervals (CI) were calculated relative to the CTP category with the biggest estimated penumbra. A two-sided p < 0.05 was considered statistically significant. Analyses were performed using SPSS (IBM Corp., Armonk, NY, USA).

## Results

We included 579 patients with acute anterior circulation LVO stroke treated with MT (mean age 70.8 years, 51.5% male). The mean onset-to-groin puncture time was 4.2 hours, 45% received IVT, and successful recanalization (TICI ≥ 2b) was achieved in 81%. Favourable 90-day outcome (mRS ≤ 3) was demonstrated in 61.9%. Visual CTP was available in 539 patients, automated CTP in 528 patients and both were available in 520 patients for agreement analysis.

### Association between visual CT perfusion (CTP) and functional outcome

Visual CTP categories demonstrated a clear shift toward higher penumbra in patients with favourable outcome (Table 1). The association was significant (χ^2^ = 29.5, p < 0.001).

**TABLE 1.** Distribution of patients according to visual CT perfusion (CTP) and outcome

Visual CTP (penumbra category)	mRS ≤ 3	mRS > 3	Total
**0–25% penumbra**	14	27	41
**25–50% penumbra**	26	27	53
**50–75% penumbra**	84	60	144
**75–100% penumbra**	215	86	301
**Total**	**339**	**200**	**539**

1 Pearson χ^2^ = 29.545, p < 0.001

### Association between automated CT perfusion (CTP) and functional outcome

Automated CTP categories were also associated with outcome (χ2 = 21.6, p < 0.001), although the strength of association was lower than for visual CTP (Table 2).

**TABLE 2.** Distribution of patients according to automated CT perfusion (CTP) and outcome

Automated CTP (penumbra category)	mRS ≤ 3	mRS > 3	Total
**0–25% penumbra**	2	7	9
**25–50% penumbra**	14	18	32
**50–75% penumbra**	71	65	136
**75–100% penumbra**	239	112	351
**Total**	**326**	**202**	**528**

1 Pearson χ^2^ = 21.562, p < 0.001

### Agreement between visual and automated CT perfusion (CTP)

Among 520 patients with both visual and automated CTP assessment, agreement between the four-level classifications was only fair (κ = 0.365; 95% CI 0.299–0.431) (Table 3).

**TABLE 3.** Distribution of patients between visual CT perfusion (CTP) and automated CTP

Automated CTP category (rows)/Visual CTP category (columns)	0–25% penumbra	25–50% penumbra	50–75% penumbra	75–100% penumbra	Row total
**0–25% penumbra**	5	1	0	0	6
**25–50% penumbra**	13	9	4	5	31
**50–75% penumbra**	21	25	60	25	131
**75–100% penumbra**	0	15	70	267	352
**Column total**	**39**	**50**	**134**	**297**	**520**

1 Cohen’s κ = 0.365 (95% CI 0.299–0.431).

### Multivariable models: visual CT perfusion (CTP)

In multivariable logistic regression, visual CTP remained an independent predictor of favourable outcome in both baseline and extended models (Tables 4A and 4B). Increasing visual penumbra demonstrated a graded, positive association with favourable outcome in both models.

**TABLE 4A.** Multivariable logistic regression including visual CT perfusion (CTP) (n = 539) for favourable outcome (mRS ≤ 3); baseline model (without CT angiography [CTA] collaterals)

Predictor	OR (95% CI)	p-value
**Age (per year)**	**0.94 (0.92–0.96)**	**< 0.01**
**ASPECTS (per point)**	**1.29 (1.09–1.51)**	**< 0.01**
**Time to groin puncture**	1.01 (0.92–1.11)	0.82
**IV thrombolysis**	0.75 (0.49–1.15)	0.18
**TICI < 2b**	**0.31 (0.18–0.54)**	**< 0.01**
**Visual CTP 0–25 %**	**0.22 (0.10–0.47)**	**< 0.01**
**Visual CTP 25–50 %**	**0.30 (0.15–0.62)**	**< 0.01**
**Visual CTP 50–75 %**	**0.51 (0.31–0.83)**	**< 0.01**

1 Odds ratios (OR) with 95% CI; reference category for CTP = 75–100%

1 ASPECTS = Alberta Stroke Program Early CT Score; TICI = Thrombolysis in Cerebral Infarction

**TABLE 4B.** Multivariable logistic regression including visual CT perfusion (CTP) (n = 539) for favourable outcome (mRS ≤ 3); extended model (with CT angiography [CTA] collaterals)

Predictor	OR (95% CI)	p-value
**Age (per year)**	**0.94 (0.92–0.96)**	**< 0.01**
**ASPECTS (per point)**	**1.26 (1.07–1.49)**	**< 0.01**
**Time to groin puncture**	1.01 (0.92–1.10)	0.92
**IV thrombolysis**	0.71 (0.46–1.10)	0.13
**TICI < 2b**	**0.33 (0.19–0.57)**	**< 0.01**
**Collaterals absent/minimal**	0.37 (0.07–2.10)	0.26
**Collaterals reduced**	**0.56 (0.34–0.92)**	**0.02**
**Visual CTP 0–25%**	**0.39 (0.16–0.97)**	**0.04**
**Visual CTP 25–50%**	**0.44 (0.20–0.96)**	**0.04**
**Visual CTP 50–75%**	0.64 (0.38–1.07)	0.09

1 Odds ratios (OR) with 95% CI; reference category for CTP = 75–100%

1 ASPECTS = Alberta Stroke Program Early CT Score; TICI = Thrombolysis in Cerebral Infarction

### Multivariable models: automated CT perfusion (CTP)

Automated CTP categories contributed significantly to outcome in the baseline model (Table 5A), but the association was attenuated and lost statistical significance after inclusion of CS (Table 5B). In the extended model, age, ASPECTS, recanalization success, and CS quality remained independent predictors, whereas automated CTP did not.

**TABLE 5A.** Multivariable logistic regression including automated CT perfusion (CTP) (n = 528) for favourable outcome (mRS ≤ 3); baseline model (without CT angiography [CTA] collaterals)

Predictor	OR (95% CI)	p-value
**Age (per year)**	**0.94 (0.92–0.96)**	**< 0.01**
**ASPECTS (per point)**	**1.29 (1.10–1.52)**	**< 0.01**
**Time to groin puncture**	1.01 (0.92–1.10)	0.90
**IV thrombolysis**	0.80 (0.52–1.22)	0.29
**TICI < 2b**	**0.28 (0.16–0.49)**	**< 0.01**
**CTP 0–25%**	**0.14 (0.02–0.78)**	**0.03**
**CTP 25–50%**	**0.38 (0.16–0.93)**	**0.04**
**CTP 50–75%**	**0.58 (0.36–0.94)**	**0.03**

1 Odds ratios (OR) with 95 % CI; reference category for CTP = 75–100%

1 ASPECTS = Alberta Stroke Program Early CT Score; TICI = Thrombolysis in Cerebral Infarction

**TABLE 5B.** Multivariable logistic regression including automated syngo.via (SYV) CT perfusion (CTP) (n = 528) for favourable outcome (mRS ≤ 3); extended model (with CT angiography [CTA] collaterals)

Predictor	OR (95% CI)	p-value
**Age (per year)**	**0.95 (0.93–0.97)**	**< 0.01**
**ASPECTS (per point)**	**1.26 (1.07–1.50)**	**< 0.01**
**Time to groin puncture**	1.00 (0.92–1.10)	0.98
**IV thrombolysis**	0.72 (0.47–1.13)	0.15
**TICI < 2b**	**0.28 (0.16–0.50)**	**< 0.01**
**Collaterals absent/minimal**	0.22 (0.03–1.50)	0.12
**Collaterals reduced**	**0.39 (0.24–0.63)**	**< 0.01**
**CTP 0–25%**	0.21 (0.02–2.07)	0.18
**CTP 25–50%**	0.93 (0.33–2.57)	0.88
**CTP 50–75%**	0.76 (0.46–1.27)	0.30

1 Odds ratios (OR) with 95 % CI; reference category for CTP = 75–100%

1 ASPECTS = Alberta Stroke Program Early CT Score; TICI = Thrombolysis in Cerebral Infarction

Comparisons of OR for favourable outcome (mRS ≤ 3) according to automated and visual CTP categories in multivariable logistic regression, using the same entry parameters as above, are graphically depicted in Figure 2A (without CS) and 2B (with CS).

**FIGURE 2A.**
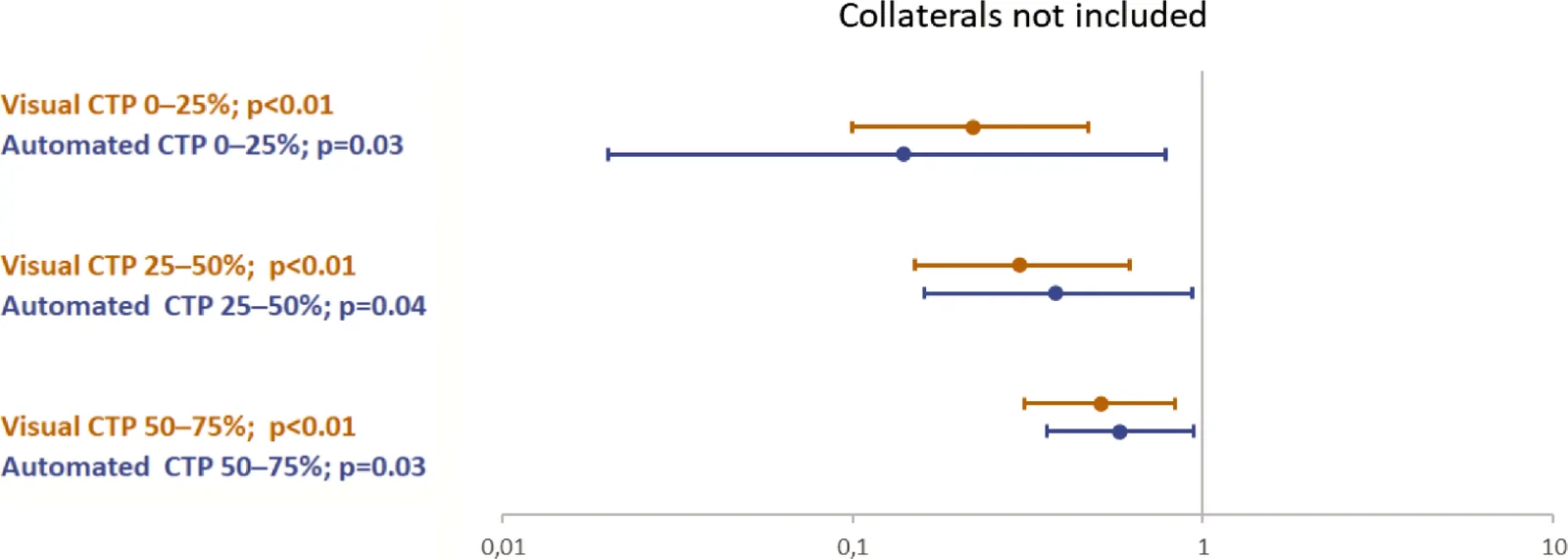
Odds ratios comparisons between automated (n = 528) and visual (n = 539) CT perfusion (CTP) for favourable outcome (mRS ≤ 3) in multivariable logistic regression; baseline model (without CT angiography [CTA] collaterals).

**FIGURE 2B.**
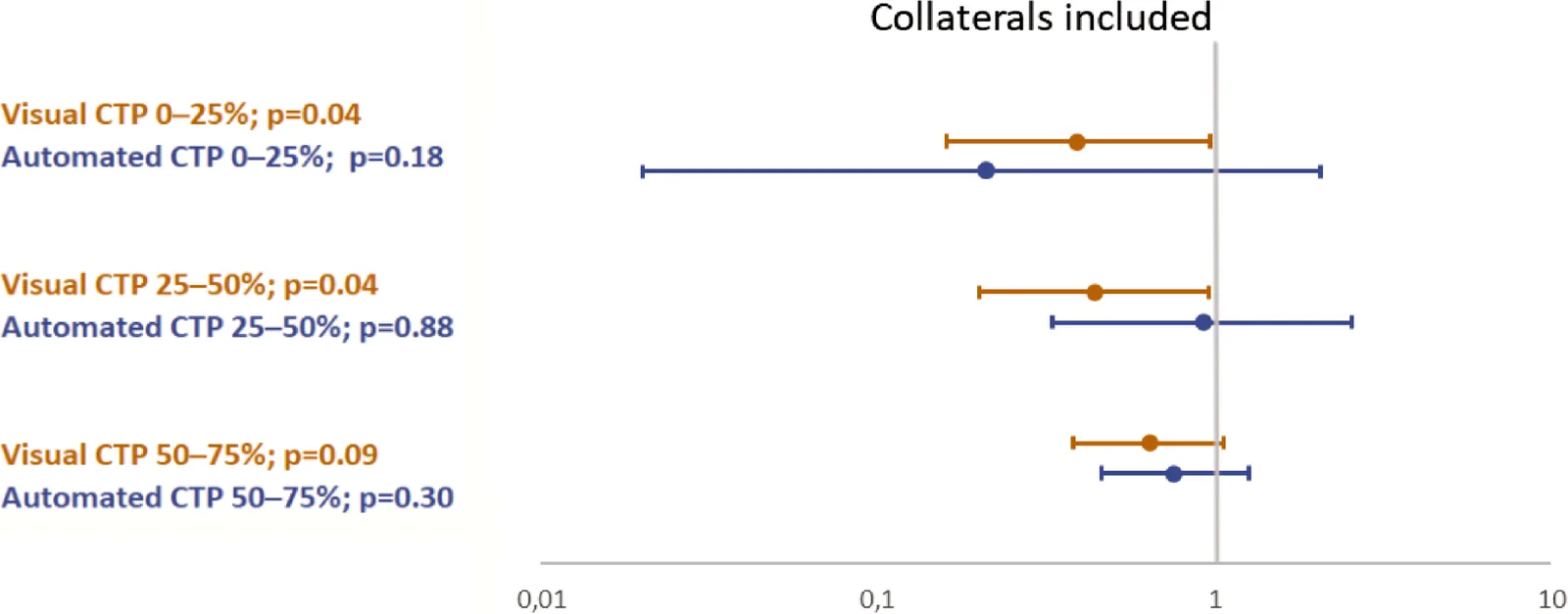
Odds ratios comparisons between automated (n = 528) and visual (n = 539) CT perfusion (CTP) for favourable outcome (mRS ≤ 3) in multivariable logistic regression; extended model (with CT angiography [CTA] collaterals).

## Discussion

In this large single-centre cohort of acute anterior circulation LVO stroke patients treated with MT, a simple visual grading of TTP maps provided prognostic information that was at least comparable to, and in adjusted models even more robust than, automated CTP analysis performed with syngo. via. Although both methods were associated with 90-day outcomes in univariate testing, agreement between them was only fair (κ = 0.36). In multivariable models, visual CTP remained an independent predictor after adjusting for age, ASPECTS, recanalization success, IVT, time to groin puncture and CS, whereas automated CTP lost significance once CS was included.

CTP has become central to treatment selection and outcome prediction in modern stroke care with major randomized trials demonstrating clinical value of perfusion-based core and penumbra estimates.^1-6^ Contemporary studies further highlight that automated CTP metrics not only predict functional independence and malignant infarction but also that their performance varies with software platforms, thresholding strategy and collateral quality.^14^

Our findings extend prior work, showing that visually identified TTP »black-hole« regions reflect severely impaired brain hemodynamics and poor prognosis.^11,12^ The only fair concordance between visual and automated CTP categories is in alignment with previous reports of substantial variability across CTP software platforms and limited spatial agreement with follow-up infarction.^7,15,16^ Several recent comparisons also suggest that visual or semi-quantitative assessment can match or outperform automated assessment for prognostication, particularly when combined with ASPECTS and CS.^12,14^ Consistent with this, visual CTP grading retained prognostic value even after adjusting for CS, whereas automated CTP did not.

CS is a dominant determinant of infarct progression and outcome.^13,17^ Its inclusion in our models reduced the predictive contribution of automated CTP but not visual CTP, possibly because visual CTP captures both the extent and severity of microvascular delay more effectively.

From a practical perspective, these findings support continued use of structured visual CTP assessment in acute stroke workflows. Visual CTP grading is rapid, universally available and independent of software specific thresholds or software versions. This is particularly relevant in centres where automated CTP may be unavailable. Moreover, visual CTP can be useful in clinical decision-making for elderly, frail or severely comorbid patients, where prognosis, not merely treatment eligibility, guides therapeutic choices.

Strengths of the study include the large real-world cohort, parallel four-category scales enabling direct comparison and multivariable models incorporating CS. Study limitations include its retrospective, single-centre design, missing CTP in a subset of patients, absence of inter-rater reliability testing for visual CTP grading and reliance on a single automated platform (syngo.via VB40). Additionally, CTP was performed with an old CT technology, which includes only four 5 mm slices through the affected area. This is in contrast with the modern stroke imaging, which these uses the whole brain CTP imaging as standard, which can influence the interpretation of CTP parameters. Different results might also emerge with alternative thresholds or newer AI-based post-processing methods.^18^

Favourable outcome (mRS ≤ 3) was used as the primary endpoint because the distribution of excellent outcome (mRS ≤ 2) within individual perfusion categories was too sparse to permit stable regression. This limitation reduces direct comparability with some major MT studies, which typically define primary endpoint by excellent outcome. Nonetheless, our study focused on the relative prognostic value of two technical perfusion assessment methods, aiming to evaluate their ability to predict the overall distribution of functional outcomes rather than selective prediction of excellent outcome alone.

Future research should validate these findings across centres, scanners, software packages and directly compare visual CTP with newer automated or AI-driven tools designed specifically for prognostication. It is likely that AI-driven tools are going to guide clinicians in the future, but simple visual CTP evaluation should remain a way of final clinical check-up before final treatment decisions.

## Conclusions

Visual CTP evaluated on TTP maps provided a slightly better prognostic performance than automated CTP evaluated by SYV using default settings. Visual CTP assessment is a simple, accessible and clinically meaningful tool for both treatment decision making and outcome prediction in anterior circulation stroke treated with MT.
